# A Specialized Peptidoglycan Synthase Promotes *Salmonella* Cell Division inside Host Cells

**DOI:** 10.1128/mBio.01685-17

**Published:** 2017-12-19

**Authors:** Sónia Castanheira, Juan J. Cestero, Gadea Rico-Pérez, Pablo García, Felipe Cava, Juan A. Ayala, M. Graciela Pucciarelli, Francisco García-del Portillo

**Affiliations:** a Laboratorio de Patógenos Bacterianos Intracelulares, Centro Nacional de Biotecnología, Consejo Superior de Investigaciones Científicas (CNB-CSIC), Madrid, Spain; b Laboratory for Molecular Infection Medicine Sweden, Department of Molecular Biology, Umeå University, Umeå, Sweden; c Centro de Biología Molecular Severo Ochoa, Consejo Superior de Investigaciones Científicas (CBMSO-CSIC), Madrid, Spain; d Departamento de Biología Molecular, Universidad Autónoma de Madrid, Madrid, Spain; Pasteur Institute

**Keywords:** *Salmonella*, cell division, intracellular pathogens, penicillin-binding proteins, peptidoglycan

## Abstract

Bacterial cell division has been studied extensively under laboratory conditions. Despite being a key event in the bacterial cell cycle, cell division has not been explored *in vivo* in bacterial pathogens interacting with their hosts. We discovered in *Salmonella enterica* serovar Typhimurium a gene absent in nonpathogenic bacteria and encoding a peptidoglycan synthase with 63% identity to penicillin-binding protein 3 (PBP3). PBP3 is an essential cell division-specific peptidoglycan synthase that builds the septum required to separate daughter cells. Since *S*. Typhimurium carries genes that encode a PBP3 paralog—which we named PBP3_SAL_—and PBP3, we hypothesized that there are different cell division events in host and nonhost environments. To test this, we generated *S*. Typhimurium isogenic mutants lacking PBP3_SAL_ or the hitherto considered essential PBP3. While PBP3 alone promotes cell division under all conditions tested, the mutant producing only PBP3_SAL_ proliferates under acidic conditions (pH ≤ 5.8) but does not divide at neutral pH. PBP3_SAL_ production is tightly regulated with increased levels as bacteria grow in media acidified up to pH 4.0 and in intracellular bacteria infecting eukaryotic cells. PBP3_SAL_ activity is also strictly dependent on acidic pH, as shown by beta-lactam antibiotic binding assays. Live-cell imaging microscopy revealed that PBP3_SAL_ alone is sufficient for *S*. Typhimurium to divide within phagosomes of the eukaryotic cell. Additionally, we detected much larger amounts of PBP3_SAL_ than those of PBP3 *in vivo* in bacteria colonizing mouse target organs. Therefore, PBP3_SAL_ evolved in *S*. Typhimurium as a specialized peptidoglycan synthase promoting cell division in the acidic intraphagosomal environment.

## IMPORTANCE

During bacterial cell division, daughter cells separate by a transversal structure known as the division septum. The septum is a continuum of the cell wall and therefore is composed of membrane(s) and a peptidoglycan layer. To date, actively growing bacteria were reported to have only a “cell division-specific” peptidoglycan synthase required for the last steps of septum formation and consequently, essential for bacterial life. Here, we discovered that *Salmonella enterica* has two peptidoglycan synthases capable of synthesizing the division septum. One of these enzymes, PBP3_SAL_, is present only in bacterial pathogens and evolved in *Salmonella* to function exclusively in acidic environments. PBP3_SAL_ is used preferentially by *Salmonella* to promote cell division *in vivo* in mouse target organs and inside acidified phagosomes. Our data challenge the concept of only one essential cell division-specific peptidoglycan synthase and demonstrate that pathogens can divide in defined host locations using alternative mechanisms.

## INTRODUCTION

Binary fission is the most common cell division mechanism known in prokaryotes. Daughter cells divide following synthesis of the division septum, a peptidoglycan (PG) structure that preserves cellular integrity during cell-cell separation. Septum synthesis is preceded by the action of numerous proteins that interact in a dynamic multiprotein complex known as the divisome (1–3). Divisome proteins assemble in a tightly defined spatial-temporal order and stoichiometry to build a ring positioned in the mid-cell, a structure referred to as the division or “Z” ring (1, 4, 5). Due to the key role played by these proteins in division, most of them are essential for bacterial life.

Although cell division has been studied mostly in model bacteria such as *Escherichia coli* and *Bacillus subtilis* (6), it has also been examined in “nonmodel” organisms, including polar flagellates, bacteria with polar (asymmetrical) growth, bacteria with multiple chromosomes, and some ecto- and endosymbionts. Representative variations of the existing models include the presence in the same bacterium of negative regulatory proteins known in Gram-positive and Gram-negative bacteria, positive regulators that position future cell division sites, dispensability of the FtsZ division protein for generating the force required for septum formation, and the existence of FtsZ-less bacteria (reviewed in reference 7).

The most accepted model of cell division involves the assembly of a ring in which division-specific PG synthases are activated to build the septum (4). Despite this detailed knowledge, how pathogens divide in the host has not yet been explored. The few existing studies, limited to pathogens grown in laboratory media or in cultured cell lines, however reveal interesting differences in cell division compared to model organisms. Thus, *Mycobacterium tuberculosis* produces daughter cells of unequal size, has FtsZ-anchoring proteins different from FtsA, and lacks a nucleoid-associated exclusion system and a Min system to prevent septum formation in erroneous locations (8). Other examples are the polarized growth and FtsZ-independent division of the intracellular bacterial pathogen *Chlamydia trachomatis* (9) and the polarized growth of the *Borrelia burgdorferi* peptidoglycan that marks new cell division sites (10).

Here, we report an unprecedented phenomenon in the nonsporulating intracellular pathogen *Salmonella enterica* serovar Typhimurium: two PG synthases capable of building the septum independently. These two PG synthases are penicillin-binding protein 3 (PBP3) and a paralog named PBP3_SAL_ that is absent in nonpathogenic bacteria. PBP3_SAL_ is a specialized PG synthase promoting cell division exclusively in acidic environments. We also provide evidence supporting a major role of PBP3_SAL_ *in vivo* in cell division following host encounter.

## RESULTS

### PBP3_SAL_ is a new peptidoglycan synthase absent in nonpathogenic bacteria and involved in cell division.

Due to its unique chemical structure not present in eukaryotes (11), peptidoglycan (PG) provides molecular patterns recognized by host defenses, a response designed to control intracellular bacterial infections. *S*. Typhimurium successfully colonizes the intracellular niche of eukaryotic cells, so we hypothesized the existence of an altered PG structure in bacteria residing inside host cells. In support of this, *S*. Typhimurium modifies PG structure inside epithelial cells to patterns not observed in bacteria grown in laboratory media (12). *S*. Typhimurium also induces inside host cells the endopeptidase EcgA, which cleaves PG stem peptides and is absent in nonpathogenic bacteria (13).

**FIG 1:**
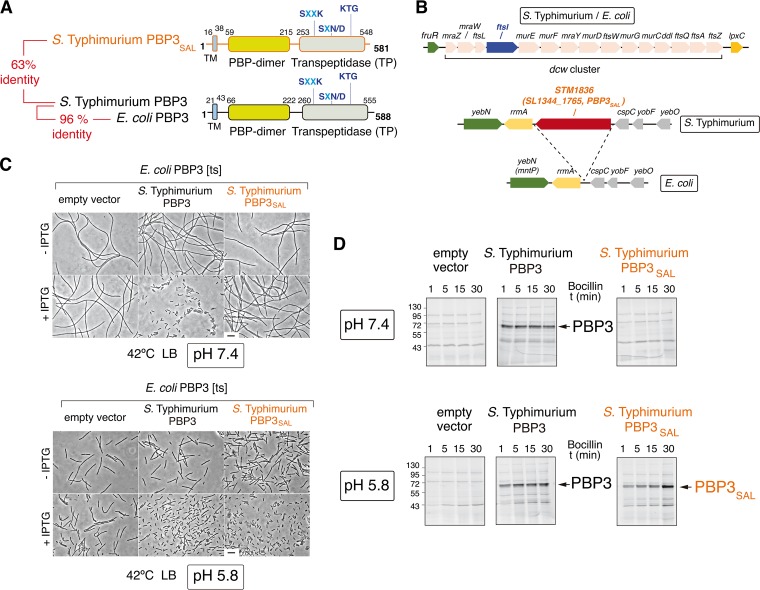
The *S*. Typhimurium PBP3 paralog (PBP3_SAL_) is a division-specific peptidoglycan (PG) synthase active at acidic pH. (A) Functional domains and catalytic motifs of PBP3_SAL_, a protein absent in nonpathogenic bacteria, compared to those of PBP3 from *S*. Typhimurium and *E. coli*. TM, transmembrane region. Numbers indicate relative positions in the protein in which the TM and the functional domains are predicted. (B) *S*. Typhimurium genome regions harboring the *ftsI* and *SL1344_1765* (*STM1836*) genes that encode PBP3 and PBP3_SAL_, respectively. The equivalent regions from the *E. coli* genome are shown, with complete synteny for *ftsI* and its flanking genes. Note that *ftsI* forms part of the *dcw* (division and cell wall gene cluster) operon and the *S*. Typhimurium *SL1344_1765* (*STM1836*) gene inserted between *rrmA* and *cspC*, encoding a predicted 23S rRNA-methyltransferase and a cold shock protein-like protein, respectively. (C) At acidic pH (5.8), PBP3_SAL_ restores division at 42°C in an *E. coli ftsI*(Ts) mutant that produces a heat-labile PBP3 variant (18). Noninducing (−IPTG) and inducing (+IPTG) expression conditions are shown. Bar, 5 µm. (D) PBP3_SAL_ binds the beta‑lactam analog Bocillin-FL at acidic pH (5.8) but not neutral pH (7.4). Fluorescent assay showing Bocillin binding to membranes from *E. coli* strain RP41 [*ftsI*(Ts)] harboring an empty vector or vectors expressing *S*. Typhimurium PBP3 or PBP3_SAL_. Bocillin binding was analyzed at the pH in which bacteria were grown, either pH 5.8 or pH 7.4, as indicated. Numbers on the left of the gels refer to the molecular mass (in kDa) of the markers used. t, time.

To identify new pathogen-specific PG enzymes, we compared the genomes of *S*. Typhimurium strain SL1344 and nonpathogenic *E. coli* strain MG1655. This analysis revealed *STM1836* (*SL1344_1765*) as a gene conserved in all *Salmonella* lineages and predicted to encode a PG synthase 63% identical to penicillin-binding protein 3 (PBP3) (Fig. 1A and see Fig. S1 in the supplemental material). *STM1836* (*SL1344_1765*) is inserted between *rrmA* and *cspC*, two genes predicted to encode a 23S rRNA-methyltransferase and a cold shock protein, respectively. Apart from *STM1836* (*SL1344_1765*), this genome region is conserved in the *S*. Typhimurium and *E. coli* chromosomes (Fig. 1B).

10.1128/mBio.01685-17.1 FIG S1  Sequence homology and functional domain organization of *E. coli* PBP3 (588 amino acids [aa]) compared to *S*. Typhimurium PBP3 (588 aa) and PBP3_SAL_ (581 aa). The conserved domains of the PBP dimer (Pfam PF03717) and transpeptidase (TP) (Pfam PF00905) are shown. The catalytic motifs SXXK, SXN/D, and KTG are also indicated. TM, transmembrane region. Download FIG S1, PDF file, 2.2 MB. Copyright © 2017 Castanheira et al. 2017 Castanheira et al. This content is distributed under the terms of the Creative Commons Attribution 4.0 International license.

PBP3, encoded by the *ftsI* gene, is an essential cell division-specific PG synthase required for synthesis of the division septum (14). PBP3 of *S*. Typhimurium shares 96% identity to that of *E. coli* (Fig. 1A and Fig. S1). Interestingly, the predicted STM1836 protein has the three motifs involved in catalysis, SXXK, SXN/D, and KTG (Fig. S1) (15), intact. On the basis of these features, we renamed STM1836 PBP3_SAL_.

BLAST analyses identified PBP3_SAL_ orthologs in the *Citrobacter* and *Enterobacter* genera and in an *E. coli* strain isolated from a stool sample from a human patient (Fig. S2A). Remarkably, no PBP3_SAL_ orthologs were found in nonpathogenic bacteria with genome deposited in databases. Of interest, the phylogenetic trees of PBP3_SAL_ and PBP3 do not overlap (Fig. S2B), which supports the idea of a second copy of PBP3 arising independently during the evolution of these bacterial genera.

10.1128/mBio.01685-17.2 FIG S2  Analysis of PBP3_SAL_ orthologs present in pathogenic bacteria outside the *Salmonella* genus. (A) Alignment of PBP3_SAL_ orthologs identified using the BLASTp tool and the protein sequence of PBP3_SAL_ from *S*. Typhimurium SL1344 as a query (UniProt accession no. or entry A0A0H3NC68). The catalytic motifs SXXK, SXN/D, and KTG are indicated. The alignment was visualized using the JalView software (version 2.9.0b2). *E. coli* strain ISC11 was isolated from the stool sample from a human patient (I. Barisic, D. Mitteregger, A. M. Hirschl, C. Noehammer, H. Wiesinger-Mayr, Infect Genet Evol 27:408-417, 2014, doi: 10.1016/j.meegid.2014.08.014). (B) Phylogenetic tree of PBP3_SAL_ orthologs identified in the following species (respective UniProt entries indicated in parentheses): *Salmonella enterica* (A0A0H3NC68), *Salmonella bongori* (A0A0K0HBL7), *Citrobacter koseri* (A0A078LDE1), *Citrobacter freundii* (A0A1C3P4L3), *Citrobacter rodentium* (D2TM39), *Enterobacter hormaechei* (F5RYR7), *Enterobacter cloacae* (A0A157YXL1), and *Enterobacter cancerogenus* (A0A0A4A271). The phylogenetic tree was generated using Clustal Omega as the multiple-sequence alignment program. For the respective PBP3 orthologs, the same organisms were used as for PBP3_SAL_. The UniProt entries of these PBP3 orthologs are indicated in parentheses after the species: *Salmonella enterica* (A0A0H3N7Y6), *S. bongori* (A0A0K0H789), *Citrobacter koseri* (A0A078LIN0), *C. freundii* (A0A0D7M478), *C. rodentium* (D2TGE1), *Enterobacter hormaechei* (F5RVU1), *E. cloacae* (A0A0M2GAS9), and *E. cancerogenus* (A0A1V6MAM3). No phylogenetic analysis of the PBP3_SAL_/PBP3 from *E. coli* ISC11 was made, since the *ftsI* gene of this isolate appears as a pseudogene in databases. Download FIG S2, PDF file, 2.8 MB. Copyright © 2017 Castanheira et al. 2017 Castanheira et al. This content is distributed under the terms of the Creative Commons Attribution 4.0 International license.

Since PBP3_SAL_ is absent in nonpathogenic bacteria, we postulated a role of this protein in virulence. A hallmark of *S*. Typhimurium pathogenicity is the capacity to proliferate and survive within acidic phagosomes (16, 17). We then reasoned that PBP3_SAL_ could assist cell division in acidic environments. To test this, and at first considering that PBP3 was not genetically manipulatable, we expressed *S*. Typhimurium PBP3 and PBP3_SAL_ in an *E. coli* mutant harboring a heat-labile PBP3 [*ftsI*(Ts)]. This *E. coli* mutant grows at 42°C, but it cannot divide at this temperature and forms long filaments (18). Unlike PBP3, which restored division at 42°C at neutral pH (7.4) and acidic pH (5.8) (Fig. 1C), PBP3_SAL_ promoted cell division only when bacteria were grown at pH 5.8 (Fig. 1C). In contrast to PBP3, PBP3_SAL_ bound the fluorescent beta-lactam derivative compound Bocillin (Boc-FL) only at acidic pH (Fig. 1D). Therefore, PBP3_SAL_ evolved from PBP3 as a specialized cell division protein with activity restricted to acidic environments.

### PBP3_SAL_ promotes cell division independently of PBP3.

To dissect the role of PBP3_SAL_ in cell division, we decided to generate an *S*. Typhimurium mutant lacking the “essential” PBP3. To this aim, we designed a genetic procedure to be performed under acidic conditions, in which PBP3_SAL_ proved to restore cell division in the heterologous *E. coli* model (Fig. 1C). As the genes downstream of *ftsI* are essential (19), we used an *S*. Typhimurium strain with duplication of a large genome region that encompasses the entire “division and cell wall” gene cluster (*dcw*), of which *ftsI* is a part (Fig. 1B) (20). The *S*. Typhimurium Δ*ftsI* mutant was generated after replacement of one of the two *ftsI* alleles with an antibiotic resistance cassette, further removed to prevent polar effects on the essential downstream genes. This was followed by segregation of the *ftsI*^+^ Δ*ftsI* merodiploid by plating in acidified medium (pH 5.8) and PCR identification of Δ*ftsI* colonies (see Fig. S3 in the supplemental material).

10.1128/mBio.01685-17.3 FIG S3  Genetic procedure used to generate an *S*. Typhimurium Δ*ftsI* mutant lacking PBP3. The procedure relied on inactivation of an *ftsI* copy in an *S*. Typhimurium strain bearing a genomic duplication with end points in the *leuA* and *proA* loci (E. M. Camacho and J. Casadesús, Genetics 157:491–502, 2001). This region includes the cell division gene cluster, *dcw*, in which *ftsI* maps (Fig. 1B). The Km^r^ cassette used to inactivate one of the two *ftsI* alleles was removed to prevent polar effects on downstream genes. The genome duplication was segregated by plating on LB plates at pH 5.8. Colonies with *ftsI*^*+*^ and Δ*ftsI* alleles were identified by PCR. See the complete description in Materials and Methods. Download FIG S3, PDF file, 0.7 MB. Copyright © 2017 Castanheira et al. 2017 Castanheira et al. This content is distributed under the terms of the Creative Commons Attribution 4.0 International license.

Two Δ*ftsI* clones were characterized: Δ*ftsI-1* and Δ*ftsI-2*. Whole-genome sequencing of these two isolates ruled out compensatory mutations that could enable inactivation of the *ftsI* gene, previously considered essential. None of the few single-nucleotide polymorphisms (SNPs) identified were shared by the two isolates (see Table S1 in the supplemental material). A relevant example was an SNP mapping in *mreB*, which encodes an actin-like protein involved in maintenance of rod morphology and cell architecture (21). This SNP was however identified in only one of the two clones lacking PBP3 (Table S1). Δ*ftsI-1* and Δ*ftsI-2* mutants were therefore considered equally representative for further assays.

10.1128/mBio.01685-17.5 TABLE S1  Single-nucleotide polymorphisms (SNPs) resulting in nonsynonymous mutations identified by whole-genome sequencing (WGS) in the Δ*ftsI* null mutants and the *ftsI*^*+*^ segregant used in this study. Download TABLE S1, PDF file, 0.1 MB. Copyright © 2017 Castanheira et al. 2017 Castanheira et al. This content is distributed under the terms of the Creative Commons Attribution 4.0 International license.

**FIG 2:**
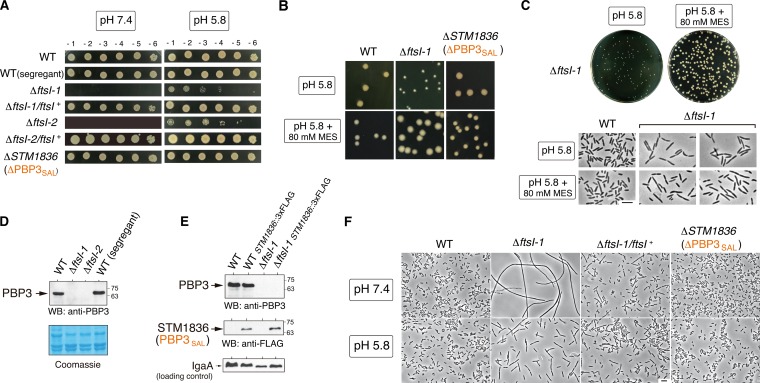
Lack of PBP3 renders *S*. Typhimurium unable to grow at neutral pH (7.4) but retaining optimal growth at acidic pH. (A) Colony-forming capacity at pH 7.4 or 5.8 of the two clones lacking PBP3 (Δ*ftsI-1* and Δ*ftsI-2*) obtained after segregation of the *ftsI*^+^/Δ*ftsI* merodiploid (see Fig. S3 in the supplemental material). Control strains include an *ftsI*^*+*^ (wild-type [WT]) segregant, the Δ*ftsI-1/ftsI*^*+*^ and Δ*ftsI-2/ftsI*^*+*^ complemented strains, and a ΔPBP3_SAL_ mutant. Numbers on the top of the upper panels refer to serial dilutions of the bacterial culture (10-fold each successive dilution). (B) Growth of *S*. Typhimurium lacking PBP3 is further stimulated in acidic pH (5.8) LB medium buffered with 80 mM HEPES. Note that in buffered medium the mutant lacking PBP3 forms colonies even larger than those of the wild type and the mutant lacking PBP3_SAL_. (C) Despite differences in colony size in nonbuffered and buffered LB solid media (pH 5.8), the Δ*ftsI-1* mutant displays similar morphology and capacity for cell division at mid-exponential phase (OD_600_ of ~0.2 to 0.3) in nonbuffered and buffered LB liquid media (pH 5.8). (D) Western blot (WB) assays confirm the absence of PBP3 in membrane fractions obtained from the Δ*ftsI-1* and Δ*ftsI-2* mutants. (E) Unaltered relative PBP3_SAL_ levels in the absence of PBP3, discarding coregulation between both proteins. The inner membrane protein IgaA (41) was used as a loading control. (F) The lack of cultivability of the Δ*ftsI-1* mutant at neutral pH (7.4) is due to a defect in cell division.

The lack of PBP3 rendered bacteria unable to grow at neutral pH (Fig. 2A), confirming that PBP3_SAL_ is active only at acidic pH. Complementation with a wild-type *ftsI* allele (PBP3) restored growth of the Δ*ftsI-1* and Δ*ftsI-2* mutants at neutral pH (Fig. 2A). In these assays, we observed that the mutants lacking PBP3 formed small colonies on Luria broth (LB) plates at pH 5.8 (Fig. 2A). Interestingly, the same mutants grew as large colonies when the acidic pH (5.8) of the plates was buffered with 80 mM MES [2-(*N*-morpholino)ethanesulfonic acid] (Fig. 2B and C). These colonies were even larger than those of wild-type bacteria or the mutant lacking PBP3_SAL_ (Fig. 2B), suggesting that bacteria using exclusively PBP3_SAL_ for cell division could be better adapted to proliferate in an acidic environment. This was further corroborated in infection assays (see below). Importantly, despite the difference in colony size found on buffered and nonbuffered acidic plates, buffering liquid LB medium (pH 5.8) with 80 mM MES did not result in major differences in the morphology or capacity of the Δ*fts-1* mutant to divide in mid-exponential phase (Fig. 2C). This was consistent with the only slight increases of ~0.2 to 0.3 pH units that were detected in nonbuffered LB medium (pH 5.8) under these growth conditions, irrespective of the bacterial strain used (data not shown). These observations therefore support the high specialization of PBP3_SAL_ for promoting cell division only in acidic environments.

As further characterization of the Δ*ftsI-1* and Δ*ftsI-2* strains, Western blot assays confirmed the absence of the “essential” PBP3 (Fig. 2D). Moreover, the lack of PBP3 did not alter PBP3_SAL_ levels (Fig. 2E), discarding coregulation or other compensatory effects between PBP3 and PBP3_SAL_. Microscopy studies evidenced that the lack of cultivability of the Δ*ftsI-1* mutant on plates at neutral pH (7.4) was due exclusively to its inability to perform cell division at this pH (Fig. 2F). We also noted that the strict dependence of acid pH for PBP3_SAL_ activity was linked to altered sensitivity to some beta-lactams compared to PBP3 (Fig. S4A and Table S2). These changes suggest that the catalytic site of both proteins might differ at the structural level. Accordingly, Boc-FL binding assays showed that PBP3_SAL_ has reduced affinity for some beta-lactam antibiotics that are avidly bound by PBP3, as is the case of cefuroxime (Fig. S4B to D).

10.1128/mBio.01685-17.4 FIG S4  PBP3_SAL_ shows reduced affinity for beta-lactam antibiotics. (A) Etest-based antibiotic susceptibility assays showing increased Δ*ftsI-1* mutant resistance to the cephalosporin cefuroxime. All strains were grown on LB plates at pH 5.8. (B and C) Bocillin binding assays performed at pH 5.8 with cefuroxime as a competitor show the lower PBP3_SAL_ affinity for this antibiotic compared to PBP3. (D) Specific inhibition of cell division by 1 µg/ml cefuroxime in wild-type bacteria but not in Δ*ftsI-1* bacteria confirms reduced binding of this beta-lactam antibiotic to PBP3_SAL_. Bacteria were grown in LB at pH 5.8 to mid-exponential phase (OD_600_ of ~0.2 to 0.3). Bar, 5 µm. Download FIG S4, PDF file, 2.4 MB. Copyright © 2017 Castanheira et al. 2017 Castanheira et al. This content is distributed under the terms of the Creative Commons Attribution 4.0 International license.

10.1128/mBio.01685-17.6 TABLE S2  Antibiotic susceptibility profile of *S*. Typhimurium strains lacking PBP3 or PBP3_SAL_. Download TABLE S2, PDF file, 0.1 MB. Copyright © 2017 Castanheira et al. 2017 Castanheira et al. This content is distributed under the terms of the Creative Commons Attribution 4.0 International license.

PBP3_SAL_ therefore can promote cell division independently of PBP3, until now considered an essential division protein. Our data also imply that PBP3_SAL_ is weakly inhibited by beta‑lactam antibiotics in wide clinical use that target preferentially PBP3.

### PBP3_SAL_ production is tightly regulated by acid pH.

**FIG 3:**
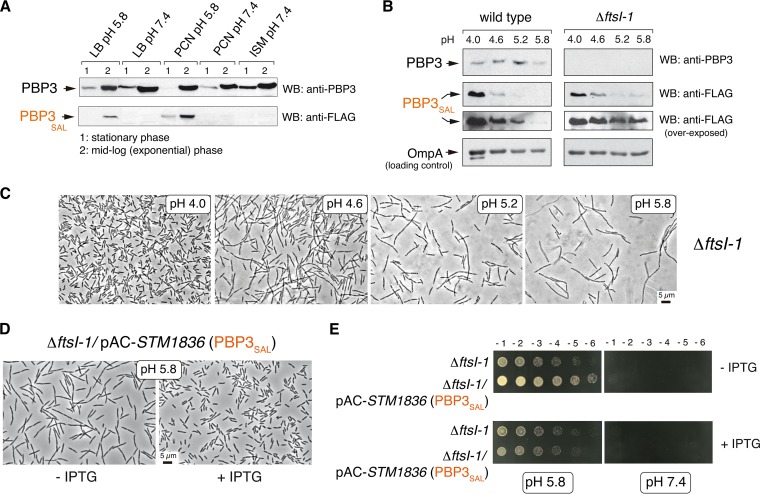
The amount of PBP3_SAL_ produced by *S*. Typhimurium controls cell division in acidic environments. (A) Relative levels of PBP3 and PBP3_SAL_ detected by Western blotting (WB) in total protein fraction of *S*. Typhimurium strain MD2559 [*STM1836*(*PBP3*_*SAL*_)::3×FLAG] grown at the indicated pH and growth phases in different laboratory media (LB, PCN [47], and ISM [48]). Note that the production of PBP3_SAL_ is stimulated in acidified media. The results shown are representative of three independent experiments. (B) The relative levels of PBP3_SAL_ increase as the pH of the media drops in the range of 5.8 to 4.0. Total protein fractions prepared at exponential phase (OD_600_ of ~0.2 to 0.3) in LB medium at the indicated pH values are shown. The outer membrane protein OmpA was used as a loading control. The results shown are representative of three experiments. (C) Cell division rate in the Δ*ftsI-1* mutant increases at lower pH values in concordance with higher levels of PBP3_SAL_. Note the gradual reduction in cell size as the pH of the medium decreases. (D) Increased cell division rate of the Δ*ftsI-1* mutant at pH 5.8 resulting from ectopic expression of PBP3_SAL_ from plasmid. Note the reduction in cell size in inducing conditions (+ IPTG). (E) An increase in PBP3_SAL_ relative levels does not restore cell division in neutral pH, supporting the specialization of PBP3_SAL_ to function only in acidic environments. Numbers on the top of the upper panels refer to serial dilutions of the bacterial culture (10-fold each successive dilution).

To determine how *S*. Typhimurium regulates PBP3_SAL_ expression, we used an *STM1836* (*PBP3*_*SAL*_)::3×FLAG-tagged strain. PBP3_SAL_-3×FLAG is fully competent for cell division since such construction was possible in a Δ*ftsI* background (Fig. 2E). *S*. Typhimurium expresses PBP3_SAL_ only in acidic (pH 5.8) nutrient media (Fig. 3A), unlike PBP3.

The *Salmonella*-containing phagosome can reach pH values of ≤4.5 (16, 17). On the basis of this evidence, we monitored PBP3_SAL_ production at pH values below 5.8. In contrast to PBP3, which remained at constant levels at all pHs tested, the amount of PBP3_SAL_ increased significantly as the pH of the medium dropped, with the highest levels detected at pH 4.0 (Fig. 3B). This regulation was observed regardless of the presence or absence of PBP3 (Fig. 3B). Consistent with the increased amount of PBP3_SAL_ in acidified media, bacteria producing only PBP3_SAL_ displayed a gradual reduction in cell size as pH decreased (Fig. 3C). Thus, the amount of PBP3_SAL_ influences the rate at which *S*. Typhimurium divides in acidic environments. This was further supported by the reduction in cell size in mildly acidic pH (5.8) medium linked to overexpression of PBP3_SAL_ (Fig. 3D), a change however not sufficient to restore division at neutral pH (Fig. 3E). Therefore, PBP3_SAL_ promotes cell division exclusively in acidic environments, with the division rate under these conditions directly related to the amount of PBP3_SAL_ that is produced by the pathogen.

### PBP3_SAL_ governs *S*. Typhimurium cell division inside eukaryotic cells.

**FIG 4:**
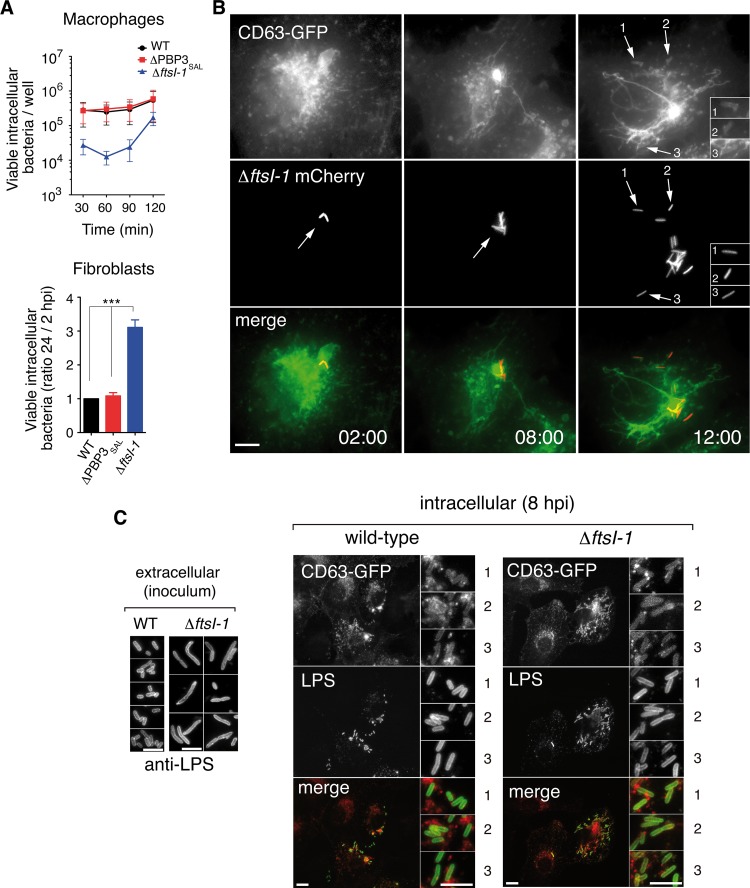
PBP3_SAL_ promotes division of intracellular *S*. Typhimurium. (A) Intracellular proliferation rates of isogenic mutants lacking PBP3 or PBP3_SAL_. Note the higher proliferation rate observed for the Δ*ftsI-1* mutant defective in PBP3. Data are the mean ± standard error (error bars) from three independent experiments. Values that are significantly different (*P* < 0.001) are indicated (***). (B) Time-lapse microscopy of live cells shows proliferation of intracellular Δ*ftsI-1* bacteria in fibroblast phagosomes. Transfected NRK-49F fibroblasts stably expressing the phagosomal membrane glycoprotein CD63 (34) were used. Images correspond to the same infected fibroblast, acquired at the indicated times (in hours) postinfection. The arrows and numbers (1 to 3) indicate different intracellular bacteria within phagosomal compartments. Bar, 10 µm. (C) Restoration of normal cell size in the Δ*ftsI-1* mutant when bacteria colonize the acidic intraphagosomal environment in fibroblasts. Bars, 5 µm (extracellular), 10 µm (intracellular wild-type and Δ*ftsI-1* strains), 5 µm (insets for wild-type and Δ*ftsI-1* strains). The numbers 1, 2, and 3 in the insets for intracellular wild-type and Δ*ftsI-1* strains show different fields of the same image.

To assess the roles of PBP3 and PBP3_SAL_ in the phagosomal acidic environment, mutants lacking the respective PG synthase were used to infect cultured cells. We used macrophages, since they are preferentially targeted by *Salmonella in vivo* (22), and fibroblasts, in which persistence and proliferation of this pathogen within phagosomal compartments have been extensively investigated (23). Bacteria producing only PBP3_SAL_ divided efficiently in the intracellular environment, with bacterial loads slightly higher than wild-type bacteria or the mutant producing only PBP3 (Fig. 4A). However, the lack of PBP3_SAL_ did not affect intracellular proliferation (Fig. 4A), compatible with a more versatile activity of PBP3 capable of promoting cell division at neutral and acidic pH (Fig. 1C).

Live-cell imaging microscopy confirmed that PBP3_SAL_ alone supports *S*. Typhimurium division inside phagosomes (Fig. 4B). Interestingly, bacteria producing only PBP3_SAL_, which showed a slightly larger cell size than wild-type bacteria in the inoculum (LB medium at pH 5.8), had similar average cell sizes once the bacteria proliferated inside the eukaryotic cell (Fig. 4C). This cell size reduction suggested that intracellular *S*. Typhimurium could divide inside host cells with an intraphagosomal pH lower than 5.8. Therefore, PBP3_SAL_ promotes *S*. Typhimurium cell division efficiently inside phagosomes of cultured eukaryotic cells.

### PBP3_SAL_ is induced by *S*. Typhimurium *in vivo.*

**FIG 5:**
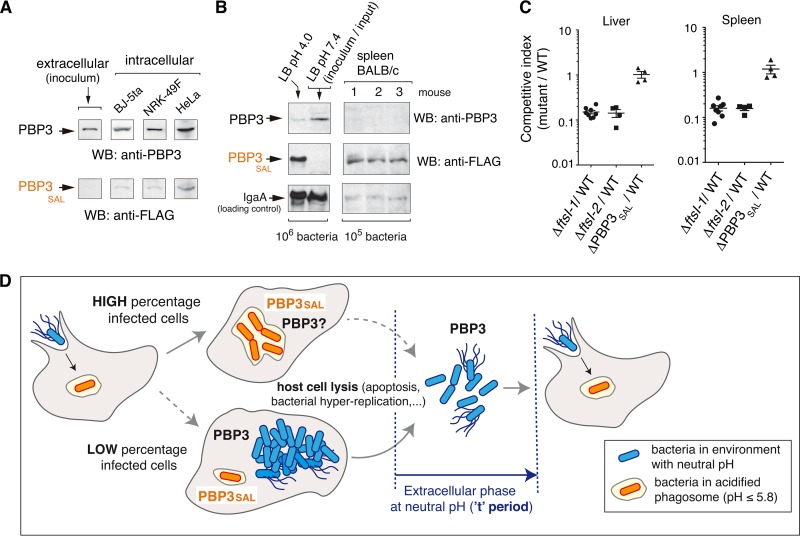
*S*. Typhimurium increases PBP3_SAL_ production inside eukaryotic cells and *in vivo* in mouse organs. (A) Relative levels of PBP3 and PBP3_SAL_ detected in intracellular bacteria (*PBP3*_*SAL*_::3×FLAG-tagged strain) after infection of BJ-5ta human fibroblasts (24 hpi), NRK-49F rat fibroblasts (24 hpi), and HeLa human epithelial cells (16 hpi). Extracellular bacteria used to infect the cells (inoculum grown in LB at pH 7.4) were used as a control. The results shown are representative of three independent experiments. (B) PBP3_SAL_ production increases at higher levels than PBP3 *in vivo*. The relative levels of both proteins in spleen extracts from three BALB/c mice challenged intraperitoneally with the *PBP3*_*SAL*_::3×FLAG-tagged strain are shown. (C) Competitive index between isogenic mutants lacking either PBP3 or PBP3_SAL_ and wild-type bacteria following intraperitoneal challenge of BALB/c mice. Liver and spleen extracts were obtained at 48 h postchallenge. (D) Model depicting the contribution of PBP3 and PBP3_SAL_ to *S*. Typhimurium cell division in different host environments: intracellular (neutral pH cytosol or acidic phagosomes) and extracellular sites during transit between infected cells. Note that the absence of PBP3 can impair normal progression of the infection, since bacteria necessarily pass an extracellular stage in a neutral pH environment (referred to here as the “t” period). A long “t” period could result for Δ*ftsI* bacteria in long filamentous cells (Fig. 2F), unable to infect new nearby host cells. In contrast, the versatile PBP3 could promote division in the phagosomal environment in the absence of PBP3_SAL_.

The *in vitro* assays performed in cultured cell lines did not discriminate the extent at which PBP3 and/or PBP3_SAL_ are used by wild-type bacteria inside host cells. Western blot assays detected both PBP3 and PBP3_SAL_ in bacteria obtained from infected-cell cultures (Fig. 5A). Unlike PBP3, which is detected in both extra- and intracellular bacteria, PBP3_SAL_ is produced *de novo* by intracellular bacteria (Fig. 5A). Strikingly, BALB/c mouse infection assays revealed that *S*. Typhimurium produces *de novo* much larger amounts of PBP3_SAL_ than PBP3 when it colonizes target organs such as the spleen (Fig. 5B). These data are consistent with PBP3_SAL_ playing an important role in *S*. Typhimurium division following host encounter.

Competition experiments in mice revealed, however, that the lack of PBP3, but not that of PBP3_SAL_, resulted in virulence attenuation (Fig. 5C). This result is nonetheless consistent with the specialized role of PBP3_SAL_—active only in acidic environments—and therefore unable to replace PBP3 outside host cells. In contrast, PBP3 functions at both acidic and neutral pH (Fig. 1C) and, therefore, is capable of replacing PBP3_SAL_ in any episode during host colonization. These views are combined in the model depicted in Fig. 5D. Our model considers PBP3_SAL_ as the key cell division protein used by *S*. Typhimurium facing the acidic intraphagosomal environment. Conversely, PBP3 could contribute to cell division in environments with neutral pH, such as the cytosol of infected cells or extracellular locations transiently occupied by the pathogen during successive rounds of infection. This extracellular phase, which we call the “t” period, might be critical if only PBP3_SAL_ is produced, since bacteria could not divide outside cells, acquiring a filamentous morphology (Fig. 2F). This dramatic increase in cell size could impede infection of bystander host cells.

## DISCUSSION

This study provides the first evidence of two peptidoglycan (PG) synthases capable of building independently the division septum in a nonsporulating bacterium. The data obtained for *S*. Typhimurium contrast with the redundancy in cell division recently claimed for two PG synthases of *B. subtilis* (24). In this organism, a PG synthase involved in cell elongation can rescue division in a mutant harboring a catalytically inactive PBP2B, the cell division-specific synthase. Unlike what our study demonstrates for *S*. Typhimurium PBP3, the deletion of PBP2B is lethal in *B. subtilis* (25). There is also a precedent in *E. coli* of a penicillin-binding protein, PBP6b, which displays higher activity at acidic pH and plays a major role in maintaining the cell shape at low pH (26). Unlike PBP3_SAL_, whose production is strictly restricted to pH values that are ≤5.8 (Fig. 3A and B), PBP6b expression by *S*. Typhimurium is favored at acidic pH albeit also detected in minimal PCN medium (47) at neutral pH and in actively growing bacteria in LB medium (G. Rico-Pérez, M. G. Pucciarelli, and F. García-del Portillo, unpublished data). This differs with the absence of production of PBP6b reported in LB medium at pH 7.5 (26). Slight differences in the regulation exerted by the two bacteria on PBP6b might explain such distinct expression profiles.

Our data are consistent with two PG synthases involved in *S*. Typhimurium cell division with one of them, PBP3_SAL_, highly specialized to function in acidic environments. The versatility of PBP3, capable of ensuring infection in mice alone as shown by the ΔPBP3_SAL_ mutant, raises the question of why *S*. Typhimurium acquired PBP3_SAL_ as the preferred PG synthase to be produced by this pathogen *in vivo* (Fig. 5B). *S*. Typhimurium infects the host alternating intracellular episodes with extracellular stages in which bacteria contact cells to initiate new infection cycles (22). Despite PBP3 being capable of promoting division inside and outside cells, our *in vitro* assays—in which extracellular bacteria are killed with gentamicin—demonstrate that PBP3_SAL_ is more effective in promoting division inside host cells. PBP3_SAL_ expression is also tightly regulated, and this can allow the pathogen to fine-tune the division rate in distinct host cell types or infection stages. This view of different cell division events inside and outside host cells is supported by the reduction of the *Listeria monocytogenes* division period occurring inside host cells (27). An early study of *Shigella flexneri* also identified a small hydrophobic protein, IspA, whose inactivation impaired division in intracellular bacteria (28). In contrast to PBP3_SAL_ however, IspA is present in nonpathogenic *E. coli*, and its exact function remains unknown.

Our study shows that the PBP3_SAL_/PBP3 ratio is much higher *in vivo* than in *in vitro* using cultured cells (compare Fig. 5A and B). Importantly, both infection models support *de novo* synthesis of PBP3_SAL_, which was expected as PBP3_SAL_ levels respond to acidification of the media (Fig. 3B). The augmented production of PBP3_SAL_ at low pH values such as pH 4.0 agrees with studies of bone marrow macrophages—with intraphagosomal pH estimated to range from pH 4.0 to 4.5—showing that this acidification is essential for survival of intracellular *S*. Typhimurium (17). Our findings therefore support the idea of PBP3_SAL_ as a specialized division protein contributing to the adaptation of *S*. Typhimurium to the intracellular lifestyle. As PBP3_SAL_ exhibits lower affinity for beta-lactam antibiotics that bind to PBP3 with a high affinity (see Table S1 of supplemental material), new drugs targeting this enzyme capable of promoting division independently of PBP3 inside host cells should be searched.

Since PBP3_SAL_ orthologs are present in *Citrobacter* spp. and *Enterobacter* spp., it will be of interest to dissect whether these proteins also evolved to promote cell division in host acidic intracellular environments. In fact, some species of these genera are pathogenic and reported to invade nonphagocytic cells and survive and proliferate within macrophages (29, 30).

The genetic procedure described in this study allowed us to generate an *S*. Typhimurium mutant lacking the hitherto considered essential gene *ftsI*, encoding PBP3. To our knowledge, there is no precedent for this inactivation strategy, specifically designed to delete a gene encoding an “essential” cell division protein. A requirement in the case of PBP3 was an intermediate step involving duplication of *ftsI* and the downstream genes within the *dcw* operon. Without the Δ*ftsI* mutant, we had been unable to discover the role that PBP3_SAL_ plays in cell division. In this respect, it is worth referring to a recent study of *Pseudomonas aeruginosa* reporting the inability to knock out the cell division-specific PBP3 (31), even considering the presence in this organism of a PBP3 homologue named PBP3x (32). Testing different infection-related conditions (different pHs and biofilm states) may offer the opportunity of deleting PBP3 and discerning whether PBP3x could, as PBP3_SAL_ in *S*. Typhimurium could, be a specialized protein with an important role in the pathogen-host interaction.

Collectively, this study therefore expands our view of the strategies used by *S*. Typhimurium to adapt to host environments, highlighting the dichotomy existing between extracellular and intracellular episodes taking place in the host and how cell division has probably been programmed to occur differently in these phases. A major challenge for future studies will be to understand what the benefits of PBP3_SAL_ are compared to PBP3 in promoting pathogen division inside the acidic phagosome.

## MATERIALS AND METHODS

### Bacterial strains and plasmids.

Bacterial strains and plasmids used in this study are listed in Table S3 of supplemental material. Bacteria were grown routinely at 37°C in Luria broth (LB) at pH 7.4 adjusted with 1 M NaOH. The Δ*ftsI* mutants and derivative strains were grown in the LB medium adjusted with 1 M HCl to pH values of 5.8 or lower (range of 4.0 to 5.8). For defined assays, the LB medium (pH 5.8) was buffered with 80 mM MES [2-(*N*-morpholino)ethanesulfonic acid]. When necessary, ampicillin (100 µg/ml), kanamycin (30 µg/ml), and chloramphenicol (10 µg/ml) were added to the media. PCN (47) and ISM (48) minimal media were used for some assays, as indicated.

10.1128/mBio.01685-17.7 TABLE S3  *S*. Typhimurium and *E. coli* strains and plasmids used in this study. Download TABLE S3, PDF file, 0.1 MB. Copyright © 2017 Castanheira et al. 2017 Castanheira et al. This content is distributed under the terms of the Creative Commons Attribution 4.0 International license.

### Eukaryotic cell lines and bacterial infection assays.

Human epithelial HeLa cells (ATCC CCL-2) and fibroblasts BJ-5ta (ATCC CRL-4001) and NRK-49F (ATCC CRL-1570) were used. Cells were propagated in Dulbecco’s modified Eagle’s medium (DMEM) or Eagle minimum essential medium containing 10% (vol/vol) fetal bovine serum (FBS) at 37°C in a 5% CO_2_ atmosphere as described previously (33). Murine macrophages RAW264.7 were cultured in DMEM with 4 mM glutamine and 5% FBS. RAW264.7 macrophages were activated with 7.5 ng/ml gamma interferon (IFN-γ) added 24 h before infection. Prior to infection, macrophages were incubated in fresh medium without IFN-γ. For large-scale experiments needed to monitor protein production by intracellular bacteria, eukaryotic cells were incubated in 500-cm^2^ plates as described previously (33). Bacteria used to infect eukaryotic cells (inoculum) were grown overnight at 37°C in LB at pH 7.4 or 5.8 without shaking.

### Phase-contrast microscopy.

Overnight bacterial cultures were centrifuged (8,000 × *g*, 10 min, room temperature [RT]) and diluted 1:100 in LB at the desired pH (7.4, 5.8, or in the range 4.0 to 5.8) to exponential phase (optical density at 600 nm [OD_600_] ≈ 0.2 to 0.3). To maintain stable growth conditions (exponential phase), cultures were diluted 1:3 every 40 min in LB medium at the appropriate pH (5.8 or 7.4). After 2 h, bacteria were harvested (4,300 × *g*, 5 min, RT), washed in phosphate-buffered saline (PBS), and fixed with 3% paraformaldehyde. Images were acquired on an inverted Leica DMI 6000B microscope with an automated CTR/7000 HS controller (Leica Microsystems) and an Orca-R2 charge-coupled-device (CCD) camera (Hamamatsu Photonics).

### Immunofluorescence microscopy.

Stable NRK-49F fibroblast transfectants expressing CD63-GFP (green fluorescent protein) were seeded on coverslips in 24-well plates. Cells were infected, fixed, and processed as described previously (34). Rabbit polyclonal anti-*S*. Typhimurium lipopolysaccharide (LPS) and Alexa Fluor 594-conjugated anti-rabbit were used as primary and secondary antibodies, respectively. Cells were observed with a Leica DMI 6000B wide-field microscope, and images were acquired with an Orca-R2 CCD camera.

### Live-cell imaging microscopy.

Stable transfected NRK-49F cells expressing CD63-GFP were used. Cells were cultured in μ-Slide four-well dishes (Ibidi), maintained in Fluorobrite DMEM (Thermo Fisher Scientific) supplemented with 10% FBS and 4 mM glutamine, and infected with the Δ*ftsI* strain expressing mCherry (Table S1). Images were acquired in an inverted Leica DMI 6000B microscope at 1-h intervals with multiposition mode and 2 × 2 binning as described previously (34).

### Preparation of membrane fractions and Bocillin-FL binding assay.

*Escherichia coli* strain RP41 [*ftsI*(Ts)] (18) was grown overnight at 30°C in LB at pH 5.8 or LB at pH 7.4. Cultures were diluted 1:100 in 100 ml of fresh medium, and bacteria were grown to an OD_600_ ≈ 0.2 to 0.3, when 1 mM isopropyl-β-d-1-thiogalactopyranoside (IPTG) was added. Cultures were further incubated for 2 h. Bacteria were harvested by centrifugation (4,400 × *g*, 10 min, 4°C) and washed in 0.1 M phosphate buffer (mix of mono-/disodium phosphate solutions) adjusted to pH 5.8 or 7.4. After centrifugation (12,000 × *g*, 15 min, 4°C), the cells were suspended in 20 ml of acidified or neutral phosphate buffer and lysed in a French press, and the lysates were centrifuged at low speed (4,000 × *g*, 10 min, 4°C). The supernatant was centrifuged (150,000 × *g*, 35 min, 4°C). Pellets containing membrane material were washed once, centrifuged at high speed (150,000 × *g*, 35 min, 4°C) and suspended in 1 ml of phosphate buffer (pH 5.8 or 7.4). Samples were adjusted to 0.1 mg/ml for binding assay with the fluorescent beta-lactam derivative Bocillin FL (Boc-FL) (Molecular Probes) as described previously (35).

### Ectopic expression of PBP3 and PBP3_SAL_.

*Salmonella enterica* serovar Typhimurium *ftsI* (PBP3) and *STM1836* (PBP3_SAL_) genes were cloned in the expression vector pAC-P_lac_. The *ftsI* and *STM1836* genes were amplified using primers fwSpeI-PBP3/revSpeI-PBP3 (fw stands for forward, and rev stands for reverse) and primers fwSpeI‑PBP3*/revPvuI-PBP3*, respectively (Table S4), digested with SpeI (*ftsI*) or SpeI/PvuI (*STM1836*), and ligated into pAC-P_lac_. All final constructs were sequenced to ensure that no undesired mutation was introduced during manipulation. To construct pAC-HIS::*ftsI* and pAC-HIS::*STM1836*, *ftsI* (SpeI/SpeI) and *STM1836* (SpeI/PvuI) fragments were ligated into pAC-HIS. Expression at 42°C of 6×His-*ftsI* (PBP3) and 6×His-*STM1836* (PBP3_SAL_) in *E. coli* strain RP41 [*ftsI*(Ts)] was confirmed by Western blotting using anti-6×His antibody (Clontech).

10.1128/mBio.01685-17.8 TABLE S4  Oligonucleotide primers used in this study. Download TABLE S4, PDF file, 0.1 MB. Copyright © 2017 Castanheira et al. 2017 Castanheira et al. This content is distributed under the terms of the Creative Commons Attribution 4.0 International license.

### Construction of the *S*. Typhimurium Δ*ftsI* mutant.

A P22 HT105/1 *int201* phage lysate was obtained from *S*. Typhimurium SV1604 strain (Dup [thr-469* Mu*d*P* *proA692*]), a derivative of the attenuated *S*. Typhimurium strain LT2 (36). The SV1604 strain has a duplication in the genome extending from the *thrA* locus to the *proA* locus (20). The division and cell wall (*dcw*) gene cluster (*ftsI* is one of the genes in this gene cluster) maps within this duplicate genome region. The duplication was passed by P22 HT105/1 *int201* transduction from strain SV1604 to the virulent *S*. Typhimurium SV5015 strain, generating strain MD4348 (Dup [thr-469* Mu*d*P* *proA692*]) (Fig. S3 and Table S3). Maintenance of the duplication was ensured in the presence of chloramphenicol (10 µg/ml). Phage-free transductants were identified on green plates (37). One of the two *ftsI* alleles in strain MD4348 was deleted by one-step inactivation mediated by λ-Red recombinase (38), yielding strain MD4802 (Dup [thr-469* Mu*d*P* *proA692*] *ftsI*^*+*^/Δ*ftsI*::kan). The kanamycin resistance cassette was removed using the resolvase-expressing plasmid pCP20 (39). The resulting strain was MD4805 (Dup [thr-469* Mu*d*P* *proA692*] *ftsI*^+^/Δ*ftsI*). To trigger segregation in the two alleles of the *ftsI*^+^/Δ*ftsI* merodiploid, strain MD4805 was incubated in LB (pH 5.8) without chloramphenicol for ~100 generations before plating. The resulting colonies were tested by PCR using the primers pbp3-flanking-FW (FW stands for forward) and pbp3-flanking-RV (RV stands for reverse) to differentiate the *ftsI*^*+*^ and Δ*ftsI* segregants. The procedure is depicted in Fig. S3. Other genetic procedures involving 3×FLAG tagging of chromosomal genes and one-step gene inactivation, both using λ-Red recombinase, were performed as described previously (40).

### BALB/c mouse experiments.

Intraperitoneal challenge of 8-week-old female BALB/c mice was performed as described previously (41). In competition experiments, the input mixture was 5 × 10^5^ CFU with the output determined at 48 h postinfection (hpi). Wild-type and Δ*ftsI* bacteria were differentiated by colony size using LB (pH 5.8 or pH 7.4) plates. To detect PBP3 and PBP3_SAL_ *in vivo*, mice were infected with 5 × 10^5^ CFU of the *STM183*6 (*PBP3*_*SAL*_)-3×FLAG strain, and spleen extracts were prepared at 48 hpi as described previously (41). Animal experiments adhered to the European Union principles, as established in the Legislative Act 86/609 CEE (24 November 1986), and followed the protocols established by the Royal Decree 1201/2005 of the Government of Spain. The protocols used in the study were approved by the Consejería de Medio Ambiente de la Comunidad de Madrid (permit 275/14).

### Antibodies and Western blotting.

The following antibodies were used as primary antibodies: mouse monoclonal anti-FLAG (clone M2; Sigma), mouse monoclonal anti-6×His (Clontech), rabbit polyclonal anti-IgaA (42), rabbit polyclonal anti-OmpA (gift from H. Schwarz, University of Tübingen, Germany), and rabbit polyclonal anti-PBP3 (our lab collection). Goat polyclonal anti-mouse or anti-rabbit IgG conjugated to horseradish peroxidase was used as a secondary antibody (Bio-Rad). SDS-PAGE and Western blotting were performed as described previously (33).

### Extraction of genomic DNA.

Stationary-phase bacteria were harvested by centrifugation (12,000 × *g*, 15 min, 4°C) and suspended in lysis buffer (50 mM Tris-HCl [pH 8.0], 10 mM EDTA, 100 mM NaCl, 0.2% SDS) with 4 μl RNase (10 mg/ml) (30 min, 37°C); proteinase K (20 mg/ml) was added, and samples were incubated at 65°C for 2 h. DNA was collected after three phenol:chloroform-isoamyl (2 parts of phenol to 1 part of chloroform-isoamyl) extractions, followed by precipitation at −20°C with 1/10 volumes of 3 M sodium acetate and 2.5 volumes of absolute ethanol. DNA was washed twice with 70% ethanol, suspended in 10 mM Tris-HCl (pH 8.0), and stored at 4°C.

### Whole-genome sequencing and SNP analyses.

DNA library generation and genome sequencing were conducted by Parque Científico de Madrid (http://fpcm.es/). Paired-end sequencing (2 × 150) was performed using the Illumina Miseq platform (Illumina, Inc.) to determine the whole-genome sequence of *S*. Typhimurium strains SL1344 (*hisG*), SV5015 (SL1344 *his*^+^), MD4356 (Δ*ftsI*-*1*), MD4357 (Δ*ftsI*-*2*), and MD4358 (segregant *ftsI*^*+*^) (Table S1). The average number of reads per genome was on the order of 1,270,000 (equivalent to ≈3,810,000 nucleotides) with an estimated coverage of 90× for a genome ≈ 4.2 Mb. Alignment of raw sequences was performed with BWA (43) with default parameters for paired-end reads. Samtools/bcftools (44) were used to compress, sort, index, and detect single-nucleotide polymorphisms (SNPs) from alignment results in BAM format. Finally, the biological impact of detected SNPs was determined by snpEff (45). The Integrative Genomics Viewer (IGV) browser (46) was used to visualize and to select relevant SNPs.

### Cefuroxime competition in Boc-FL binding assays.

Membrane fractions (50 µl), adjusted to a protein concentration of 0.1 mg/ml, were incubated with various concentrations of cefuroxime (0.0001 to 1.000 µg/ml) for 30 min at 37°C. An untreated sample was used as a control. The membranes were incubated with Boc-FL (30 min, 37°C) and processed as described above. These incubations were performed in phosphate buffer (pH 5.8).

### Antibiotic resistance assays.

Bacteria from overnight cultures were centrifuged (4,300 × *g*, 5 min, RT) and suspended in 1/3 of the initial volume to further spread ~200 µl in LB (pH 5.8) plates using cotton swabs. To evaluate the MIC, we used Liofilchem MIC test strips (MTS) (Werfen). MIC was determined after 18-h incubation at 37°C. To evaluate resistance to cefuroxime (CXM) in liquid culture, overnight cultures were diluted in LB (pH 5.8) to an initial OD_600_ ≈ 0.02. Cefuroxime (1 µg/ml) was added at an OD_600_ ≈ 0.2 to 0.3. After 1-h incubation with the antibiotic, bacteria were harvested by centrifugation (4,300 × *g*, 5 min, RT) and fixed with 3% paraformaldehyde to be visualized by phase-contrast microscopy.

### Statistical analysis.

Data were analyzed by one-way analysis of variance (ANOVA) using Prism version 5.0 (Graph-Pad Software). Differences in values with *P* < 0.05 were considered significant.
