# Supplementary figures and images for: A Specialized Peptidoglycan Synthase Promotes Salmonella Cell Division inside Host Cells
Source: mBio. 2017 Dec 19;8(6):e01685-17. doi: 10.1128/mBio.01685-17 (PMC5736910; doi:10.1128/mBio.01685-17)

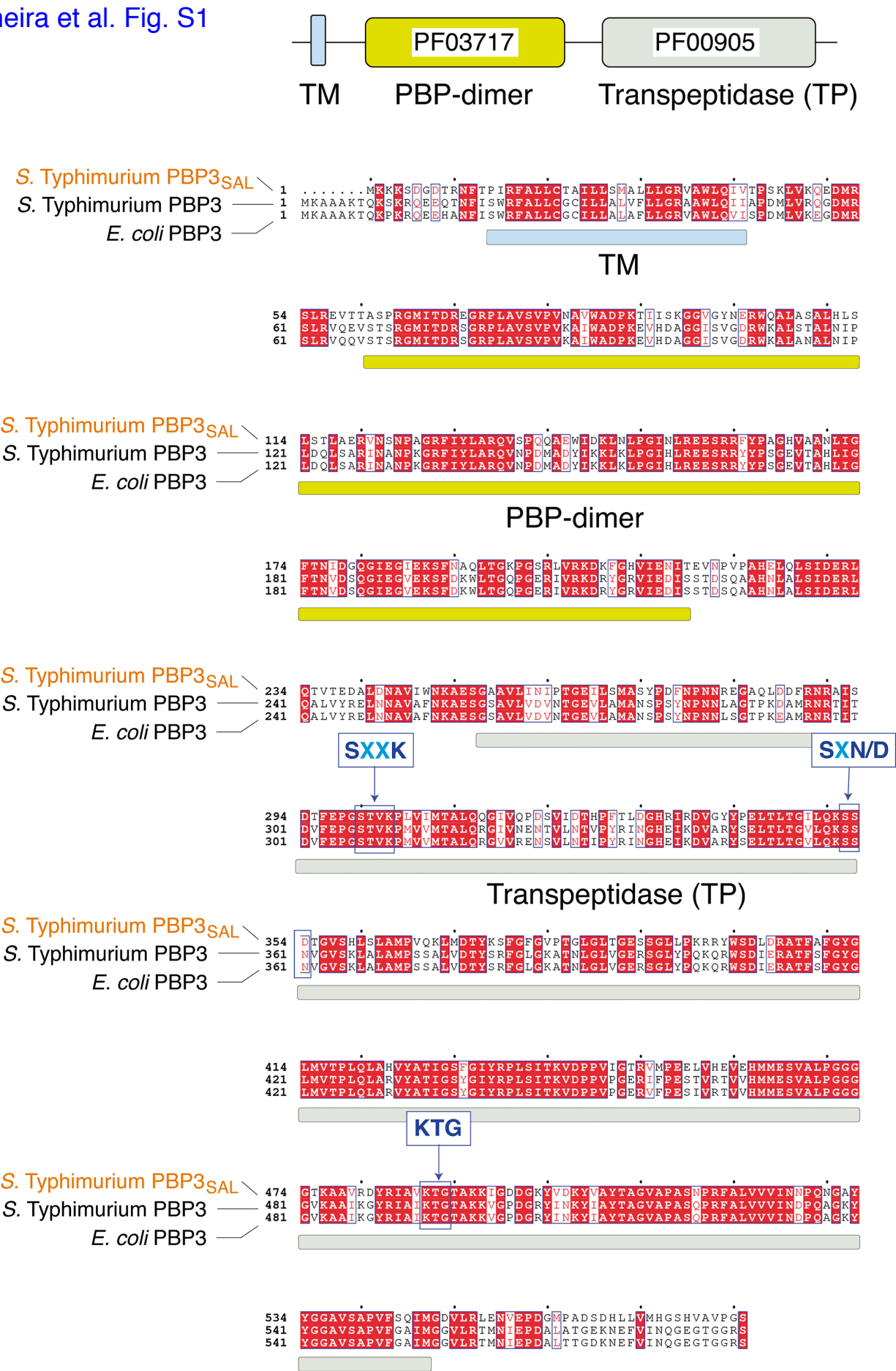

Supplement: FIG S1 [file mbo006173650sf1.pdf]

FIG S2. Castanheira et al.

A

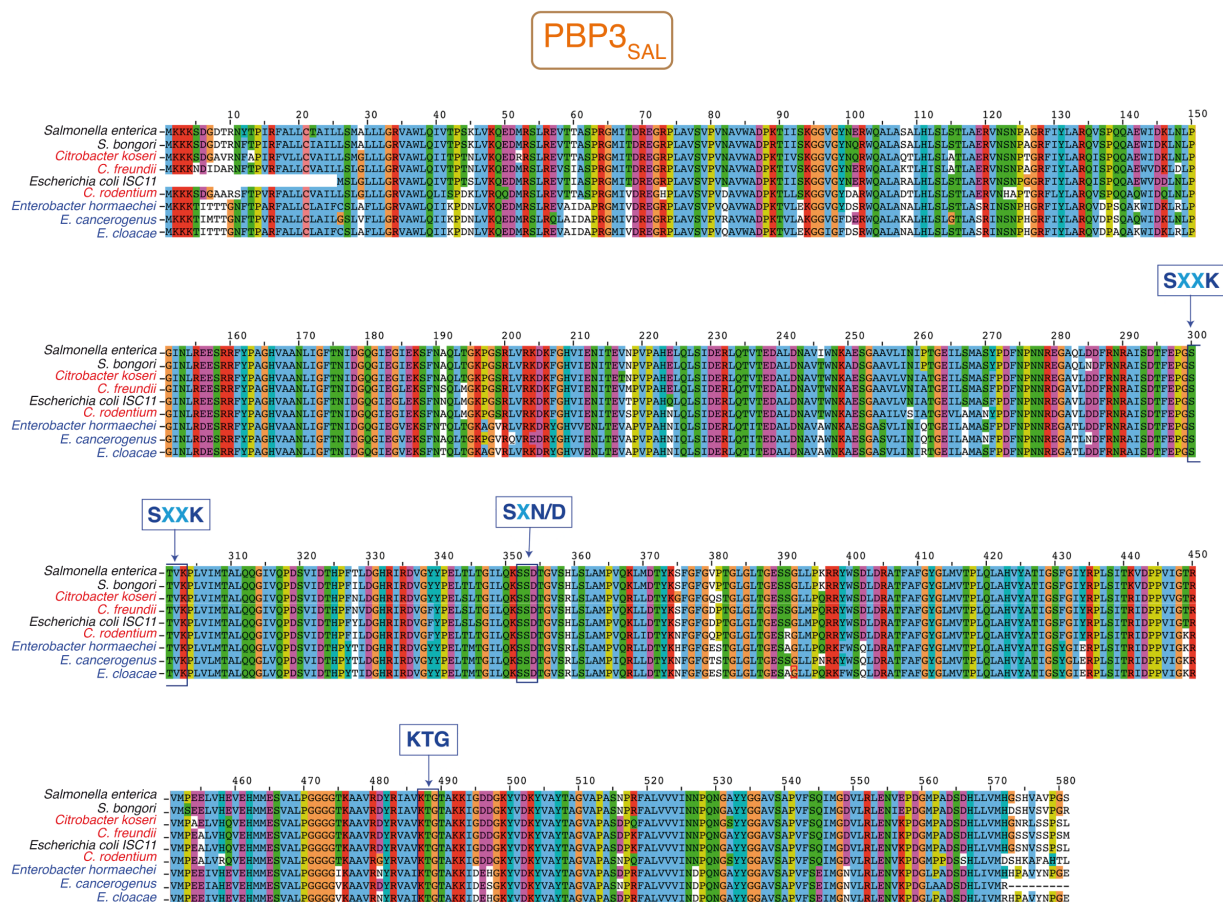

B

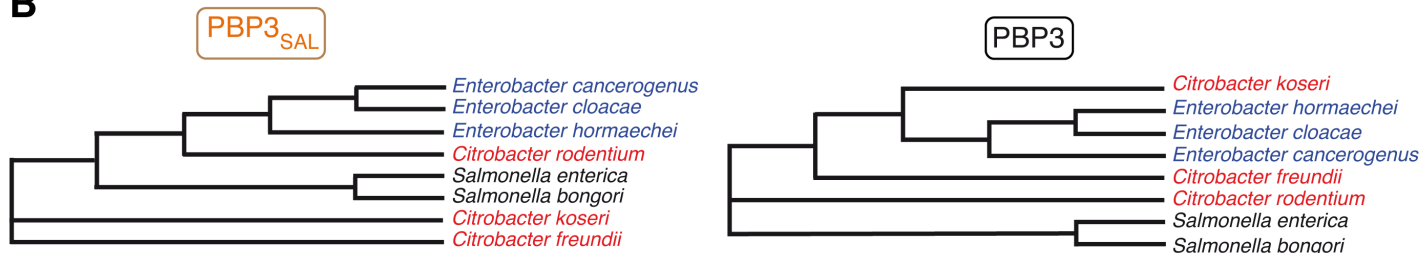

Supplement: FIG S2 [file mbo006173650sf2.pdf]

FIG S3. Castanheira et al.

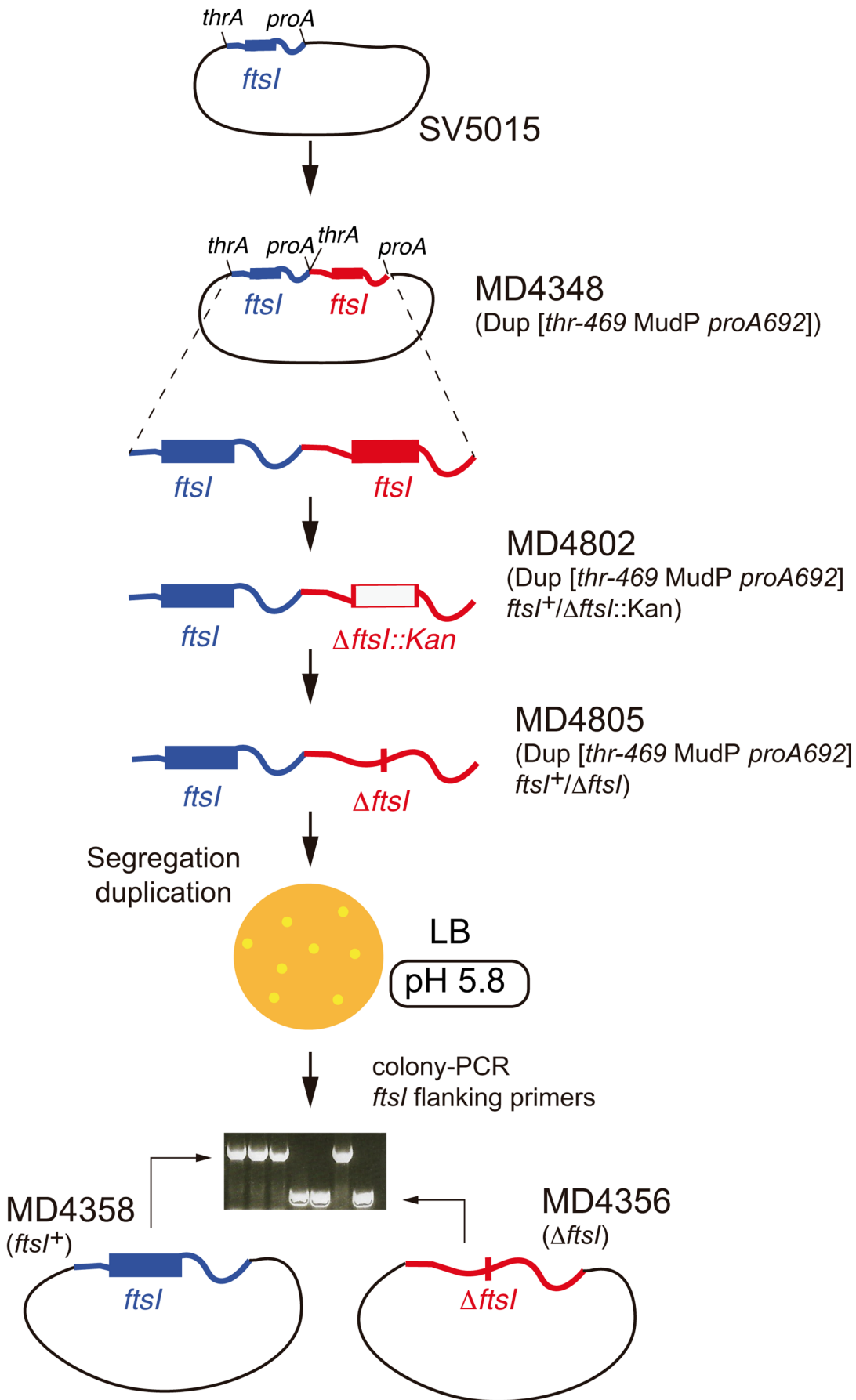

Supplement: FIG S3 [file mbo006173650sf3.pdf]

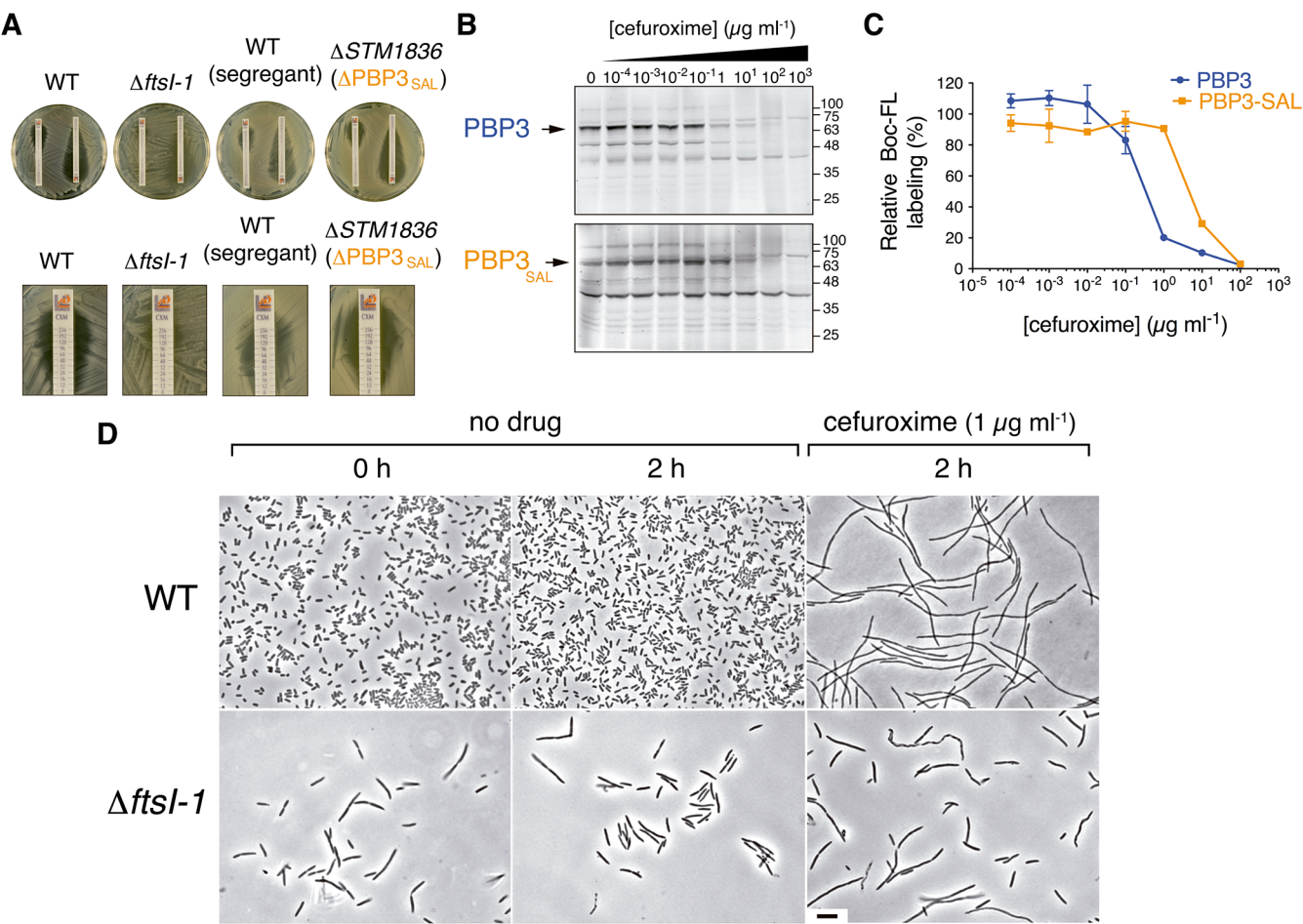

Supplement: FIG S4 [file mbo006173650sf4.pdf]
