# Supplementary material for: A Specialized Peptidoglycan Synthase Promotes Salmonella Cell Division inside Host Cells
Source: mBio. 2017 Dec 19;8(6):e01685-17. doi: 10.1128/mBio.01685-17 (PMC5736910; doi:10.1128/mBio.01685-17)
Supplement: TABLE S1 [file mbo006173650st1.pdf]

Table S1. Single-nucleotide polymorphisms (SNP) resulting in non-synonymous mutations identified by whole-genome sequencing (WGS) in the Δ*ftsI* null mutants and the *ftsI*$^{+}$ segregant used in this study. (*)

**Coordinate SNP(**)**	**Locus ID**	**Protein**	**Nucleotide/strain (†)**					**Effect**
			**SL1344 (*hisG*)**	**SV5015 (SL1344, His+)**	**MD4356 (SV5015, Δ*ftsI-1*)**	**MD4357 (SV5015, Δ*ftsI-2*)**	**MD4358 (SV5015, *ftsI*+)**	
635606	*SL1344_RS02730*	PTS mannose transporter subunit IIAB	A	T	T	T	T	Glu95Val
2135472	*SL1344_RS10615*	Thiosulphate reductase cytochrome B	G	T	T	T	T	Gly110Val
2147535	*SL1344_RS10655*	ATP phosphoribosyltransferase (HisG)	C	T	T	T	T	Pro69Leu
600714	*SL1344_RS02730*	Putative outer membrane protein	G	G	C	C	C	Val55Leu
2909450	*SL1344_RS14215*	Predicted ATP-binding protein	A	A	-	A	A	Leu278STOP
3565837	*SL1344_RS17425*	Rod-shape determining protein MreB	C	C	C	T	T	Ser48Leu
4606941	*SL1344_RS22305*	PoxA, regulator pyruvate oxidase PoxB	G	G	G	C	C	Cys89Tyr

(*) WGS confirmed endpoints of the Δ*ftsI* deletion in coordinates 141805-143342.

(**) Accession used for the SL1344 reference genome was GenBank: FQ312003.1.

(†) Strain SV5015 is the prototrophic His^{+} strain used as wild-type is this study. SV5015 was generated from the auxotrophic strain SL1344 by phage transduction (Vivero et al., 2008) and was also sequenced by WGS in this study. Strain MD4358 (SV5015, *ftsI*$^{+}$) refers to a *ftsI*$^{+}$ segregant obtained from the merodiploid strain MD4805 (*ftsI*$^{+}$/Δ*ftsI*).
