# Supplementary material for: A Specialized Peptidoglycan Synthase Promotes Salmonella Cell Division inside Host Cells
Source: mBio. 2017 Dec 19;8(6):e01685-17. doi: 10.1128/mBio.01685-17 (PMC5736910; doi:10.1128/mBio.01685-17)
Supplement: TABLE S2 [file mbo006173650st2.pdf]

Table S2. Antibiotic susceptibility profile of *S.* Typhimurium strains lacking PBP3 or $PBP3_{SAL}$ (*)

**Antibiotic (†)**	**Strain**		
	**WT**	**Δ*ftsI-1***	**Δ$PBP3_{SAL}$**
Ampicillin	0.75	1	1
Aztreonam	0.094	0.75	0.094
Cefalotin	4	8	8
Cefepime	0.19	0.19	0.19
Cefoxitin	2	6	4
Ceftazidime	0.75	0.125	0.38
Cefuroxime	8	48	12
Imipenem	0.19	0.125	0.125
Meropenem	0.023	0.016	0.032
Piperacillin	1.5	3	1.5
Kanamycin	4	6	4
Ciprofloxacin	0.023	0.064	0.032

(*) Indicated are values of minimal inhibitory concentration (MIC) (μg ml^{-1}) of a representative experiment from a total of two independent E-test assays with essentially identical results.

(†) Kanamycin and ciprofloxacin were used as control non-beta-lactam antibiotics
