# Supplementary material for: A Specialized Peptidoglycan Synthase Promotes Salmonella Cell Division inside Host Cells
Source: mBio. 2017 Dec 19;8(6):e01685-17. doi: 10.1128/mBio.01685-17 (PMC5736910; doi:10.1128/mBio.01685-17)
Supplement: TABLE S3 [file mbo006173650st3.pdf]

Table S3. *S.* Typhimurium/*E. coli* strains and plasmids used in this study

Bacterial strain/ plasmid	Relevant genotype	Source/ reference
E. coli		
MC6RP1	K-12, F$^-$, *thrA leuA proA dra drm lysA*	(García-del Portillo and de Pedro, 1990)
RP41	MC6RP1, *leu*$^+$, *ftsI (ts)*	(García-del Portillo and de Pedro, 1990)
MD4464	RP41 pAC	This study
MD4465	RP41 pAC::*STM1836*	This study
MD4471	RP41 pAC::*ftsI*	This study
MD5005	RP41 pAC-*6xHIS*	This study
MD5006	RP41 pAC-*6xHIS*-*STM1836*	This study
MD5008	RP41 pAC-*ftsI*-*6xHIS*	This study
S. Typhimurium		
SL1344	*hisG64,* virulent strain	(Hoiseth and Stocker, 1981)
SV5015	SL1344, *hisG*$^+$	(Vivero et al., 2008)
SV1604	LT2 (Dup [*thr-469**Mu*d*P**proA692*])	(Camacho and Casadesus, 2001)
MD4348	SV5015 (Dup [*thr-469**Mu*d*P**proA692*])	This study
MD4802	SV5015 (Dup [*thr-469**Mu*d*P**proA692*]) *ftsI*$^+$/Δ*ftsI*::kan	This study
MD4805	SV5015 (Dup [*thr-469**Mu*d*P**proA692*]) *ftsI*$^+$/Δ*ftsI*	This study
MD2502	SV5015 Δ*STM1836::kan*	This study
MD2559	SV5015 *STM1836::*3xFLAG-*kan*	This study
MD3842	SV5015 *STM1836::*3xFLAG-*kan ompR1009*::Tn*10*	This study
MD3895	SV5015 *STM1836::*3xFLAG-*kan slyA*::pRR10Δ*trfA*Pen^R	This study

MD3897	SV5015 *STM1836::*3xFLAG-*kan phoP7953*::Tn*10*	This study
MD4356	SV5015 Δ*ftsI-1* (segregant, clone 1)	This study
MD4357	SV5015 Δ*ftsI-2* (segregant, clone 2)	This study
MD4358	SV5015 *ftsI*$^{+}$ (segregant)	This study
MD4360	SV5015 Δ*ftsI-1 STM1836::*3xFLAG-*kan*	This study
MD4361	SV5015 *ftsI*$^{+}$ (segregant) *STM1836::*3xFLAG-*kan*	This study
MD4366	SV5015 Δ*ftsI-1* pAC-*ftsI*$^{+}$	This study
MD5023	SV5015 Δ*ftsI-1* pSEVA 237R	This study
MD5024	SV5015 Δ*ftsI-1* pAC-6xHIS-*STM1836*	This study
MD5026	SV5015 pSEVA 237R	This study
Plasmids		
pKD13	Kan^{R}, Amp^{R}	(Datsenko and Wanner, 2000)
pKD46	γ, β, exo. Amp^{R}	(Datsenko and Wanner, 2000)
pSUB11	3xFLAG sequence, Kan^{R}	(Uzzau et al., 2001)
pCP20	*FLP*$^{+}$, Amp^{R}, Cm^{R}	(Cherepanov and Wackernagel, 1995)
pAC	Cm^{R}	(Lobato-Marquez et al., 2015)
pAC-HIS	Cm^{R}	(Lobato-Marquez et al., 2015)
pSEVA 237R	Kan^{R}	(Silva-Rocha et al., 2013)
