# Supplementary material for: A Specialized Peptidoglycan Synthase Promotes Salmonella Cell Division inside Host Cells
Source: mBio. 2017 Dec 19;8(6):e01685-17. doi: 10.1128/mBio.01685-17 (PMC5736910; doi:10.1128/mBio.01685-17)
Supplement: TABLE S4 [file mbo006173650st4.pdf]

Table S4. Oligonucleotide primers used in this study

Primer	Sequence (5'-3')
fwSpeI-PBP3	CGCTACTAGTATGAAAGCAGCGGCAAAAACGCAAA
revSpeI-PBP3	CCCTACTAGTTTACGATCTGCCACCTGTTCCCTCG
fwSpeI-PBP3*	CGCTACTAGTGTGAAAAAGAAAAGCGACGGCGATA
revPvuI-PBP3*	CCCTCGATCGTTACGAACCCGGAACCGCAACGTGG
KO STPBP3 FW	AAATCGAAACGCCAGGAAGAACAGACCAACTTCATCAGTT GGCGTTTTGCGTGTAGGCTGGAGCTGCTTC
KO STPBP3 RV	CCGCCCATGATGGCACCAAATACCGGCGCGGAAACGGCGC CGCCGTAGTAATTCCGGGGATCCGTCGACC
pbp3 flanking FW	GATCCGTCCCAAGAAAATATTGTAG
pbp3 flanking RV	AGCCACCCACGGAGCAAGAAGGTCG
pAC FW	CGGCCCTCATTCGTGCGCTCTAGGA
pAC-sec	CATAATGGGGAAGGCCATCCAG
KO PBP3* Fw	GGGCGCTAAAGGGCGCAAGTGTAACGCGAATTGCGCCCCG GGAAAATCCTGTGTAGGCTGGAGCTGCTTC
KO PBP3* Rv	CCGTTTCCCGTTAAATCAATCACCTGAAAAATGATTCGGCT GGAGATCAGATTCCGGGGATCCGTCGACC
FLAG-3*-FW	TCTGGTGATGCATGGCAGCCACGTTGCGGTTCCGGGTTCGG ACTACAAAGACCATGACGG
FLAG-3*-Rv	GGGCGCAAGTGTAACGCGAATTGCGCCCCGGGAAAATCCT CATATGAATATCCTCCTTAG
FLAG STM 1836 Fw	GGCAGCTTTGGTATCTACCGTCC
FL-3* FW	TGGCGCTGTGGACATATAACG
FL-3* RV	GCCTCAGATATAAAGCCTCGCT
